# RADx Tech Viability and Steering Panels: A Model for MedTech Translational Grant Review

**DOI:** 10.1109/OJEMB.2021.3070821

**Published:** 2021-03-29

**Authors:** Paul Tessier, Michael K. Dempsey, John Collins, Steve Schachter

**Affiliations:** Consortia for Improving Medicine with Innovation and TechnologyMassachusetts General Hospital and Harvard Medical School Boston MA 02114 USA

**Keywords:** Commercialization, funding, RADx, translational research

## Abstract

RADx^SM^ Tech's mission is to rapidly accelerate deployment of SARS-CoV-2 tests and could not utilize typical grant application and review processes that can run 4 to 6 months. Instead, RADx Tech leveraged methodologies developed by CIMIT and utilized by POCTRN as described further in this special issue. RADx Tech uses a multi-stage review with two review panels, a Viability Panel and a Steering Panel, that are supported by subject matter experts and a Deep Dive team. Members of the panels have extensive commercialization and business experience in addition to scientific and technical knowledge. The Viability Panel is responsible for assessing whether the proposal is a good fit with the RADx Tech Program and whether it should be recommended to move into a Deep Dive. Less detailed information is requested in the application than a typical SBIR application since the application is refined and details added during the Deep Dive. The Steering Panel reviews the results from the Deep Dive and decides whether to recommend further funding. Everyone on the Viability Panel and Steering Panel reviews every application, thereby providing consistency and context for the reviewers. Utilization of an “assess, improve, and then select” process with review panels comprised of highly experienced review panel members has resulted in improved timing, efficiency, and effectiveness of reviews and has the potential to be extensible beyond RADx Tech.

## I. Introduction

RADx^SM^ Tech is one program within the broader Rapid Acceleration of Diagnostics (RADx) initiative that was established with a $1.5 billion investment from federal stimulus funding in April 2020. The charter of RADx Tech is to accelerate the development, validation, and commercialization of innovative point-of-care and home-based tests, as well as improvements to clinical laboratory tests, that can directly detect SARS-CoV-2, the virus that causes COVID-19, with the goal of making SARS-CoV-2 testing readily available to every American. The National Institute of Biomedical Imaging and Bioengineering's (NIBIB) Point-of-Care Technology Research Network (POCTRN), which is led by the Coordinating Center at the Consortia for Improving Medicine with Innovation & Technology (CIMIT) [1], was selected to run the extramural aspects of the RADx Tech program. CIMIT was an early pioneer in translational research with over 20 years of experience in refining translational methods, processes, and tools and had been coordinating POCTRN for over two years. The CIMIT/POCTRN methodology was the basis for the RADx Tech grant review and management processes, as described further throughout this special issue.

Expert panel reviews (Viability Panel and Steering Panel) are a key component of the RADx Tech grant review and management processes. Utilization of highly experienced industry experts from multiple disciplines is an important element in improving the timing, efficiency, and effectiveness of translational grant reviews. This is particularly true when there is an interactive application and assessment process with the applicant. An interactive model allows for new and improved processes with the ability to better assess the translational potential of the application.

According to the Translational Research Institute at the University of Arkansas for Medical Sciences, “The goal of translational research is to translate (move) basic science discoveries more quickly and efficiently into practice.” [2] For many, if not most, translational programs the path for quickly and efficiently moving into practice is through commercial efforts. Therefore, some translational granting programs such as the SBIR program have a commercialization/business focus, which is also the primary goal of RADx Tech and the reason for including industry experts with significant commercial/business experience in the RADx Tech review panels in combination with clinical and scientific experts.

## II. Traditional Translational Grant Review Background

Like most research grant programs, typical translational grant programs utilize a linear application and review process. A typical process contains the following steps.

1. Release of solicitation (request for proposals)
2. Application submission
  The applicant submits a grant application according to specific solicitation and program instructions, guidelines, and systems.
3. Application verification
  Each application is checked for completeness and/or errors.
4. First level of review (peer review)
  A peer review is conducted against all review criteria and considerations established for evaluation of applications. A premium is placed on novelty. Reviewers provide a written critique and an impact score. A summary and peer review critiques are provided to the applicant.
5. Second level of review (programmatic review)
  A programmatic review is done taking into consideration the scientific and technical merit of the proposed project as determined by peer review, availability of funds, and relevance of the proposed project to program priorities.
6. Communication of the review and decision to applicant.
  Applicants are typically allowed, if not encouraged, to contact the granting agency with their questions before submitting the application. However, there is typically minimal, if any, interaction between applicants and the funding organization between the time an application is submitted and the review process is completed. Some organizations may contact the applicant with questions during the review process. Once a review summary is provided, applicants can contact the agency to discuss the review outcome of the application and obtain guidance. If the application is not funded, the applicant can discuss how the application can be improved if it is resubmitted.

Given the minimal interaction between applicants and reviewers or the funding organization pre-review, only information provided in the application is used for the full assessment. It has been estimated that applicants for SBIR grants spend approximately 100 to 200 hours preparing and submitting an application [3]. Yet it can be challenging for applicants to provide complete and detailed information given page limits and for reviewers to have all the information they need for a thorough review. Obviously, applicants want to paint the best possible picture and there is “grantsmanship” involved in the application, further complicating the review process.

Reviewers for the first level of review (peer review) are frequently comprised mostly of individuals who have expertise in relevant scientific disciplines and current research & development areas. In recent years, the make-up of review panels has included more individuals with commercialization and business knowledge, however the make-up of panels can vary significantly from panel to panel and granting organization to granting organization. The second level of review is frequently done by an advisory board who consider the input from the first level of review and assess the programmatic fit of the application.

Translational grant review cycles vary depending on the granting organization; however, they typically run in the range of 4 to 6 monhts [4], [5].

## III. RADx Tech Grant Review Process

RADx Tech's mission from the start was to rapidly accelerate deployment of SARS-CoV-2 tests and so it could not wait 4 to 6 months after receiving a proposal to initiate work. Instead of the typical process that selects projects based on reviews of what is written (“evaluate and select” process), RADx Tech employs an “assess, improve, and then select” process. After an initial assessment, RADx Tech assigns a team to work with the applicant to improve the application (called a Deep Dive; see article by Dempsey *et al.* [7] in this special issue). Results from the Deep Dive are then used to recommend and select the projects with the highest chance of success.

The application for RADx Tech is tailored to the “assess, improve, and then select” process. Less detailed information is requested in the application than a typical SBIR application since the application is refined and details added during the Deep Dive. The application contains:

• Description and characterization of the applicant organization
• Overview of the team and environment
• Solution overview and status including preliminary data and expected/target specifications
• Regulatory overview
• Development and implementation plan
• Overview of partnerships and/or collaborations
• Key risks and mitigations
• Support requested from RADx Tech
• High-level schedule and budget.

A final plan, schedule, and budget are not required since it is anticipated that elements of the plan will likely change and be refined during the Deep Dive process.

**Fig. 1.**
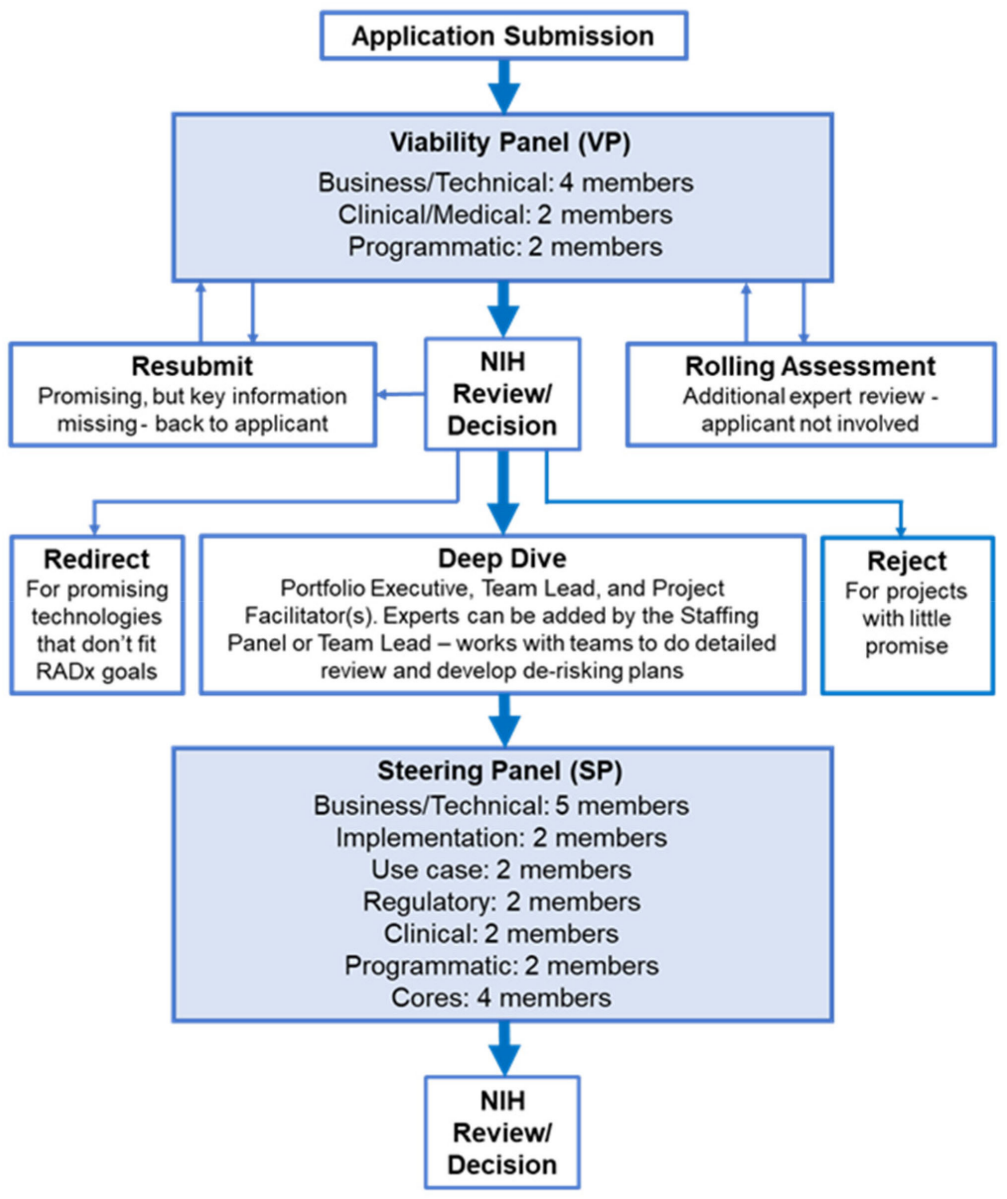
RADx Tech Review Process.

RADx Tech uses a multi-stage review process with two review panels, a Viability Panel and a Steering Panel, that are supported by subject matter experts (SMEs) and the Deep Dive team. Fig. 1 below outlines the RADx Tech review process. Applications are reviewed on a rolling basis as they are submitted. Everyone on the Viability Panel and Steering Panel reviews every application, thereby providing consistency in the reviews and context for the reviewers (there had been only two changes in personnel in the VP panel during its period of work). This process contrasts with typical peer review where there is a primary reviewer and one or two secondary reviewers, but the whole panel does not review each application in detail.

The Viability Panel is responsible for assessing whether the proposal is a good fit with the RADx Tech Program and whether it should be recommended to move into a Deep Dive. There are eight (8) members on the panel with three key areas of expertise:

• Business/technical (four panel members): All members have extensive business and technical expertise. All have served in senior technical and management roles in large companies and/or start-up operations.
• Clinical/Medical (two panel members): Both members are MDs with many years of clinical, managerial, and business experience, including in retail clinic settings.
• Programmatic (two panel members): Both members have managerial positions in the funding organization (NIH).

Members of the Viability Panel all have more than a decade of experience, many with 30 or more years of experience.

The Steering Panel reviews the results from the Deep Dive and decides whether to recommend further funding (WP1 or WP2 – see article by Dempsey *et al.* [7] in this special issue). There are nineteen (19) members on the panel across six key areas of expertise:

• Business/Technical (five panel members): All members have extensive business and technical expertise having served in senior technical and management roles in large companies and/or start-up operations.
• Implementation (two panel members): Both members have vast experience scaling operations and implementing clinical testing programs nationally.
• Use case/clinical (two panel members): Both members have wide-ranging experience in clinical settings and translational research.
• Regulatory (two panel members): Both members have many years of experience obtaining regulatory approvals and working with regulatory agencies.
• Clinical (two panel members): Both members have extensive experience in clinical testing.
• Programmatic (two panel members): All members have managerial positions in the funding organization (NIH).
• RADx Tech Cores (four panel members; see articles by Nehl *et al.* [8], Gibson *et al.* [9], and Walsh *et al.* [10] in this special issue): All members have advanced degrees and extensive clinical and academic experience in investigating and evaluating medical technologies.

The eight members of the Viability Panel also serve on the Steering Panel, thereby bringing continuity to the review process while also bringing in fresh perspectives. As with the Viability Panel, the members of the Steering panel each have more than a decade of experience, many with more than 30 years of experience.

The Deep Dive is conducted by a RADx Tech team comprised of a highly experienced Portfolio Executive, Team Lead, and Project Facilitators who are supported by SMEs as needed (see article by Dempsey *et al.* [7] in this special issue). The Deep Dive team works closely with the applicant to review, refine, and further assess the application over a 1- to 2-week period, involving many hours of interaction with the applicant and their team. The Deep Dive team plays a critical role of adding an expert and objective assessment of an applicant team's status and potential but does not vote whether to move a project into WP1 or WP2 – that is the responsibility of the Steering Panel. Additional insights are thus provided while allowing the Steering Panel review to be independent and objective.

The breadth and depth of experience of the Viability Panel and Steering Panel members allows for rich and detailed discussion of projects against the review criteria during facilitated panel meetings. Across the panel members there is extensive knowledge and therefore discussion on science, technologies, companies, characteristics of successful company leaders, use models and users, regulations, manufacturing, and distribution.

## IV. Viability Panel

**Fig. 2.**
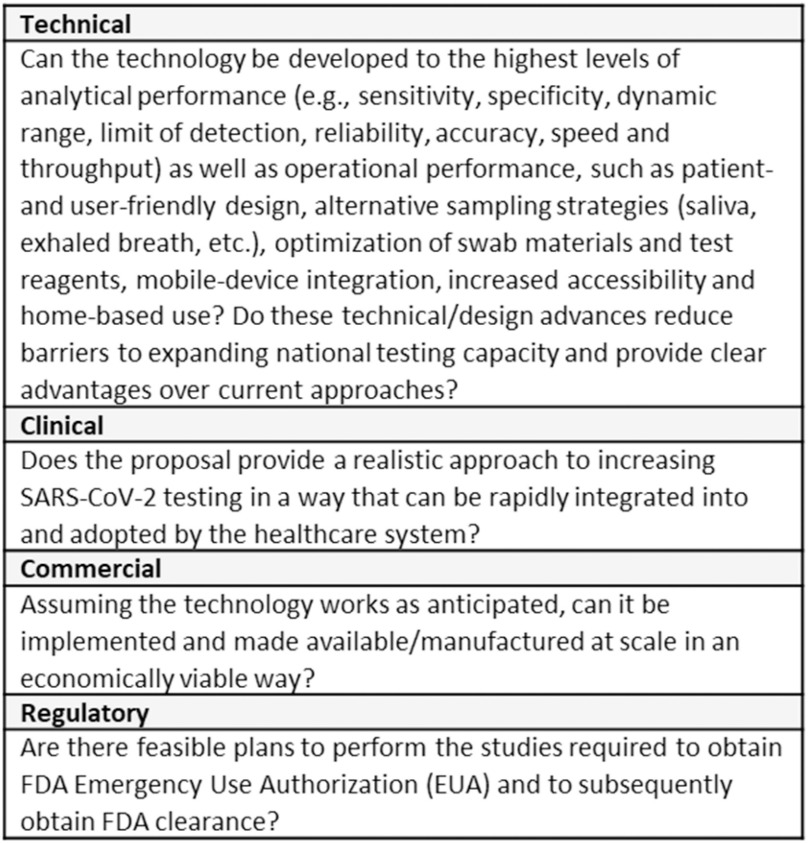
RADx Tech Project Review Criteria.

The Viability Panel assesses an application against the RADx Tech project review criteria outlined in the RADx Tech solicitation (Fig. 2) taking into account the fit with different use case(s) for the product. Anticipated use environments included healthcare settings such as doctor's offices and pharmacies, manufacturing facilities, offices, schools, and universities as well as more traditional testing locations like hospitals and labs. Use case fit is a significant factor in the assessment of applications.

Using an online system known as CoLab (see article by Collins *et al.* [11] in this special issue), each member reviews all the applications independently and completes an evaluation form that includes a recommendation and rationale for the recommendation. Reviewers have the options to recommend:

1) Moving the application to Deep Dive
2) Requesting the applicant Resubmit with additional information
3) Sending the application to a Rolling Assessment
4) Redirecting the application to another funding mechanism
5) Rejecting the application.

After each member conducts their independent review, the Viability Panel meets as a group to reach a consensus on a recommendation to the NIH. For applications that seem meritorious but are lacking information, the Viability Panel can request that the applicant resubmit the application with clarifications and/or additional information in specific areas requested by the Viability Panel. Resubmitted applications are reviewed using the same process outlined above and are typically re-reviewed within 48 hours of being resubmitted. In instances where the Viability Panel feels an application contains adequate information, but the Viability Panel could use additional expert review, the application is forwarded to a Rolling Assessment. Rolling Assessments are conducted by SMEs selected with specific expertise to address the questions posed by the Viability Panel. Based on the assessment received from the SMEs, the Viability Panel discusses the application and makes a recommendation to the NIH. The Viability Panel can make one of three recommendations; move the application to Deep Dive, redirect the application to another funding mechanism, or reject the application. Viability Panel members can change their comments and personal recommendation in CoLab after the panel discussions, but they are not encouraged to “harmonize” their vote with the consensus of the group. Rather, they are encouraged to maintain their individual perspectives and opinions, if they persist after the discussions, so that NIH could take in the perspectives of all the panel members when making their decisions.

Applications are reviewed by the Viability Panel as they are received, typically within two days of submission, and the NIH decision to reject, redirect, or advance to Deep Dive is typically done within a week of the Viability Panel recommendation. The objective of the Viability Panel review is to ensure that only meritorious projects advance to Deep Dive and to do so as quickly as possible. The ability to request a resubmission from the applicant and to direct an application to a Rolling Assessment provides rapid gathering of additional information enabling a fast, efficient, and effective review.

The Viability Panel has served as a model for a panel that was formed to review applications for another NIH-funded program that was established about 8 months after RADx Tech launched to support projects that were redirected from RADx Tech for another funding mechanism. Many of the people on the Viability panel also serve on this panel.

## V. Steering Panel

The Steering Panel reviews the results from the Deep Dive and decides whether to recommend further funding. During the Deep Dive, the RADx Tech Deep Dive team works closely with the applicant to review and refine the technology and development plans all the way through to commercialization. On average, a Deep Dive takes approximately fourteen (14) days. The goal of the Deep Dive is to assess the project for possible advancement into one of two funding stages known as RADx Work Packages 1 and 2 (WP1 and WP2). WP1 is similar to SBIR Phase 1 and WP2 is similar to SBIR Phase 2 and 3 combined. The Deep Dive team assesses the project based on the same assessment criteria used by the Steering Panel and in addition assesses the capabilities of the applicant team and their willingness to work closely with RADx Tech experts.

**Fig. 3.**
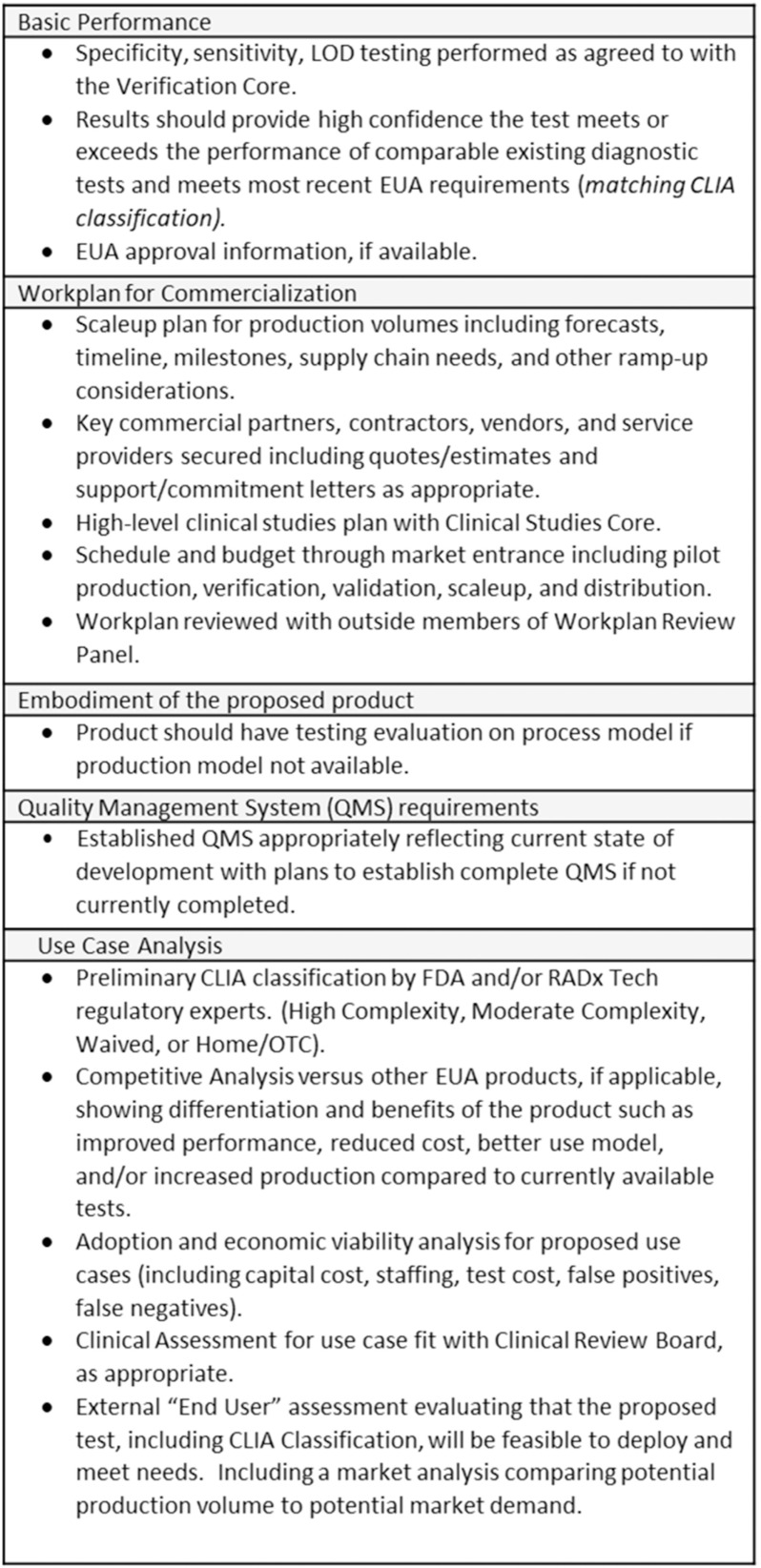
Criteria for Advancement to WP2.

In addition to the previously outlined assessment criteria (Fig. 2), the Deep Dive team and Steering Panel are asked to assess whether the proposed work for WP1 will provide sufficient evidence to justify proceeding forward to WP2 and whether the proposed WP2 plan is appropriate for bringing the product to market. For highly advanced projects, the Deep Dive team and/or Steering Panel can recommend that the project go directly to WP2. The criteria for advancing directly to WP2 are the same as for advancing from WP1 to WP2 (see Fig. 3).

For evaluation of projects being considered for WP2, the Deep Dive team is assisted by RADx Tech groups (“Cores” and panels) with specific areas of expertise, as described in detail elsewhere in this special issue. These groups provide assessment and assistance including benchtop performance verification (Clinical Validation Core), clinical studies planning (Clinical Studies Core), work plan development (Workplan Review Panel), and usability and use case fit (Clinical Review Panel). Assessment of the proposed Clinical Laboratory Improvement Amendments (CLIA) classification and Emergency Use Authorization (EUA) plan are also provided by the FDA and/or RADx Tech regulatory experts.

Once the Deep Dive is complete, the Deep Dive team uploads summary information and a summary presentation into the on-line CoLab system and schedules a review meeting with the Steering Panel. Steering Panel members review the material prior to the review meeting and presentation. The Team Lead from the Deep Dive presents the results of the Deep Dive to the Steering Panel - the applicant is not present for the presentation so that the facilitated discussion can be frank. There is a question-and-answer period followed by a recommendation vote by the Steering Panel. Non-voting members of the Cores and NIH personnel participate as observers on the Steering Panel and contribute to the discussions. Panel members have the options to recommend:

1) Moving the application to WP1
2) Moving the application directly to WP2
3) Redirecting the application to another funding mechanism
4) Rejecting the application.

As with the Viability Panel, Steering Panel members can change their comments and personal recommendation so that NIH could take in the perspectives of all the panel members when making their decisions.

Steering Panel reviews can typically be scheduled within a week and the Steering Panel recommendation to the NIH is made at the meeting. The subsequent NIH decision to reject, redirect, or advance to WP1 or WP2 is typically done within a week receiving the Steering Panel recommendation.

## VI. Results to Date

As of the writing of this article, 703 applications have been reviewed through the Viability Panel, of which 138 moved into Deep Dives and Steering Panel reviews (approximately 20%), and 60 of 138 Deep Dive projects have advanced to WP1 or WP2 (44%). The average times for the various stages of the process are as follows:

• Within one business day from completed application submission to Viability Panel review
• Nine days from application submission to NIH decision on Viability recommendation
• Fourteen days for Deep Dive completion
• Thirty-five days from application submitted to NIH decision on Steering Panel NIH recommendation (final funding decision).

The time to a funding decision for RADx Tech of 35 days compares very favorably to other translational granting programs; e.g., the NIH SBIR submission deadline to estimated award date is approximately 6 months [4], and the NSF SBIR process typical takes 4 to 6 months [5]. Using these timeframes as a reference, the RADx Tech review process saved between 13 and 24 years of cumulative delay time in starting projects across the 60 RADx Tech funded projects.

In a survey, RADx Tech applicants reported an average effort of 53 hours to complete the application. It has been estimated that NIH SBIR/STTR grants may require up to 100–200 hours of work in total to prepare. [2] The NSF states the time per response, including the time for reviewing instructions, averages 120 hours. [6] Compared to the effort required for these application efforts, the RADx Tech application process saved between 23 and 34 person-years of application effort across the 703 applications submitted (assuming 150 hours average for an NIH SBIR, 120 hours for an NSF SBIR, and 2000 working hours in a person-year).

Recruiting highly experienced industry experts for reviewers on the Viability Panel and Steering Panel was not difficult, ironically, because of the pandemic, and people were anxious to help in these unprecedented times. Likewise, the reviewers were willing to work on weekends and holidays when needed to keep the process moving as rapidly as possible. The time commitment of reviewers varied widely depending on which panel(s) they participated on and the stage of the RADx Tech program. At times, it was not unusual for a Viability Panel member to dedicate over 40 hours a week to the program and to do so for multiple consecutive weeks.

## VII. Discussion and Conclusion

Based on the above-described results and savings, it seems reasonable to conclude that an “assess, improve, and then select” process utilizing highly experienced reviewers provides a fast, efficient, and effective review methodology and has benefits over the typical “evaluate and select” process. Keys to the RADx Tech process are expert facilitation to improve the application (through Deep Dive), availability of SME and assistance of Cores and panels, and the use of highly experienced industry experts for reviewers.

Ultimately, it would be very informative to compare business results such as rate of successful commercialization, average time to market, and revenue generated for the RADx Tech program compared to other commercialization-focused translational grant programs. As of the writing of this article, 12 RADx Tech supported projects have achieved Emergency Use Authorization and are shipping more than 500,000 tests per day. This is an early indication that the RADx Tech process is highly effective at facilitating, selecting, and accelerating projects to the marketplace.

While it is probably not reasonable to expect this level of participation in all translational granting programs, the “assess, improve, and then select” methodology utilizing highly experienced reviewers is generally transferrable.

The RADx Tech model is unique in many ways, including:

• Emphasis on speed and merging best academic and business practices; both in grant processes themselves and the need to get an innovation on the market.
• Ongoing interactions between the RADx Tech faculty and the applicants.
• Clearly defined milestones and frequent checkpoints with diverse reviewers.
• The ability to scale support up or down (including cancelling) for a given project.
• The availability of non-financial support of applicants (e.g., FDA, clinical samples, QMS).

These characteristics allowed 703 projects to be reviewed and 60 projects funded, supported and some concluded over 9 months. These projects have thus far resulted in over 500,000 tests/day. While the COVID-19 pandemic created unprecedented urgency for more testing, and the resources provided by NIBIB were large, RADx Tech provided a replicable model for how other grant programs might be managed.

For example, a RADx Tech-like process could be applied to SBIR applications. The Deep Dive would allow funding bodies to assess the likelihood of commercial success of the innovation beyond what is written in the application. Then the same Deep Dive team could follow the SBIR project, potentially on a quarterly basis, and scale up or down support as appropriate. This would allow funding to be redirected from projects that are not working out to others that are making great progress. If this happened on an on-going basis (rather than the 3 application deadlines each year), there could be a pipeline of projects with increased probability of success, all at different phases.

Another possibility is to apply the RADx Tech model to large, “moonshot” challenges. A call could be put out to solve a particular problem, and a team of business, clinical and technical experts on the topic could be formed. This team would work intimately with the various applicants to maximize the probability of the projects’ success in solving the challenge.
